# Supplementary material for: Unveiling the psychological traits of multi-marathoners: Insights from TIPI personality trait analysis
Source: PLoS One. 2025 Apr 1;20(4):e0311647. doi: 10.1371/journal.pone.0311647 (PMC11961011; doi:10.1371/journal.pone.0311647)
Supplement: Attachment — Submitted filename: Rebuttal TIPI Final 30.10.2024.docx [file pone.0311647.s001.docx]

**Editor: PLoS One**

***Title of the article:*** *Unveiling the Psychological Traits of Multi-Marathoners: Insights from TIPI Personality Trait Analysis.*

**Rebuttal document**

*Thank you for the comprehensive comments from the editor and reviewers for this submission. We can certainly understand why these comments were made and we have taken them all on board and have made substantial changes to the submission based on this feedback.*

*In general, we found comments supportive of the submission and believe that this study is important not only to academics but also to the professionals and participants that engage in the sport of multi-marathoning.*

**Journal requirements**

*This revision of the submission meets PLOS ONE’s style requirements as outlined by PLOS ONE.*

**Data Availability statement**

*The entire data has been uploaded 30.10.2024 as a supplementary file to this submission. This data is anonymised and can be used to replicate the results as presented in the submission.*

**Author affiliation**

*All authors’ affiliations reflect the institution where the study was carried out.*

**Editors’ comments**

**1) The lack of theoretical foundation - the theoretical framework needs to be more developed and include some potentially relevant theories such as Goal Setting theory**

We appreciate the editor’s comment regarding the need for a more developed theoretical foundation. In this revised submission, we address this concern directly and have made the necessary substantial revisions to address this concern.

Goal Setting Theory and the Big Five Personality model are theoretical frameworks that are already incorporated in this submission and are introduced at length with appropriate references and examples in the **Introduction** and form an integral part of the **Discussion** and **Conclusion** sections. In response to the editor’s suggestion, we have enhanced the theoretical foundation further by incorporating **Self-Determination Theory (SDT)** as an additional theoretical framework, which enhances our exploration of **motivation** (both intrinsic and extrinsic). The inclusion of SDT allows us to draw connections between the **motivational aspects of multi-marathoners** and the **personality traits** measured by TIPI, further enriching the study’s theoretical depth. This new theoretical foundation consisting of The Big Five Personality Model, Goal Setting Theory and SDT are now discussed at length (with relevant references) in the **Introduction**, and all are incorporated in the **Discussion** and in the **Conclusion** to highlight how these frameworks complement each other and elucidate the way the theoretical foundation is used in the analysis of the data.

Specifically, we have incorporated the following to the **Introduction**, **Discussion** and **Conclusion** sections with associated relevant references.

**Introduction**

Another valuable framework for understanding multi-marathoners’ motivations is **Self-Determination Theory (SDT)**, which highlights how both intrinsic and extrinsic factors drive participation. In the context of multi-marathoning, SDT suggests that participants may be motivated by a combination of intrinsic factors, such as the desire for personal achievement, enhanced well-being, and social engagement, as well as extrinsic factors, such as peer recognition within the multi-marathon community. These motivational drivers can be seen as closely aligned with the personality traits measured by TIPI.

For example, individuals high in conscientiousness, a trait associated with goal-directed behaviour and self-discipline are more likely to engage in long-term training regimens that align with both intrinsic and extrinsic goals. Their high levels of goal orientation may support the pursuit of intrinsic motivations such as personal achievement, while extrinsic motivations like recognition and community approval may further reinforce their persistence in endurance sports. In contrast, extraversion, another trait measured by TIPI, may drive social engagement and peer interaction, linking directly with SDT's emphasis on relatedness as a fundamental need.

Further, SDT’s intrinsic motivation, such as the desire for personal growth and proficiency, resonate with the concept of openness to experience, a personality trait characterised by a willingness to embrace challenges and new experiences. Multi-marathoners who score high in openness may be motivated by intrinsic rewards related to exploring their limits and seeking personal growth through the physical and mental challenges of multi-marathoning.

**Discussion**

Self-Determination Theory (SDT) further enriches these findings by explaining the underlying motivations driving multi-marathoners’ commitment. SDT proposes that individuals are motivated by both intrinsic factors, such as personal achievement, proficiency, and well-being, and extrinsic factors, such as recognition and social belonging. In this study, traits like conscientiousness align with SDT’s intrinsic motivations, suggesting that multi-marathoners persist not only because of their ability to set and pursue goals but also due to internal drives such as the desire for personal growth and fulfilment. At the same time, extraversion and agreeableness may be linked to extrinsic motivations, where social connection and peer recognition play key roles in sustaining their engagement.

The integration of SDT with the Big Five and Goal Setting Theory provides a more comprehensive understanding of the psychological factors that drive the unique persistence and motivation observed in multi-marathoners.

**Conclusion**

By applying the Big Five Personality Theory, Goal Setting Theory, and Self-Determination Theory (SDT), the research provides insights into the psychological mechanisms that influence the performance and well-being of multi-marathoners. This understanding helps to guide the development of targeted interventions, including structured training programs, goal-setting strategies, and stress management resources to support both the physical and mental health of these athletes.

**2) The issue related with the measurement of Big Five Personality traits - that is using its short form that has not been validated before. This could be tackled by using the longer form on a smaller target population, or by validating the short form on any population. I recognize that doing that may require more time, but if possible (and you can ask for the extension of time needed for the revision), I would recommend you to do that. Alternatively, you need to provide a very strong rebuttal regarding this issue.**

We appreciate the Editor’s feedback regarding the use of a short form to measure Big Five personality traits. In response, we have made significant revisions to address this concern.

While longer forms, such as the 240-item NEO Personality Inventory or the 44-item Big Five Inventory, would enhance internal consistency, their length is impractical for online surveys. These instruments take 15 to 45 minutes to complete, which would likely lead to increased respondent fatigue and lower response rates, significantly affecting the statistical power of the study. In contrast, shorter forms like the **Ten-Item Personality Inventory (TIPI)** are ideal for large-scale internet-based studies, where brevity is critical.

Short-form measurement instruments have been validated extensively. For example, **Gosling et al.** (2003) validated TIPI using a dataset of over 300,000 respondents. Additionally, short forms such as the **Mini-IPIP**, **BFI-S**, and **BFI-10** have been validated in large datasets such as the **German Socio-Economic Panel (SOEP)**, a longitudinal study with over 20,000 participants (Rammstedt & John, 2007; Specht et al., 2011). These studies demonstrate that short forms can provide reliable assessments of the Big Five traits in large, diverse populations.

This trade-off between brevity and internal consistency is well-documented in the Literature, and we have updated the **Introduction** section of the manuscript to reflect this argument with appropriate references. Given the scope and scale of our study, we believe TIPI was the most practical choice.

While longer forms, such as the 240-item NEO Personality Inventory or the 44-item Big Five Inventory (24, 25) , would enhance internal consistency, their length is impractical for online surveys. These instruments take 15 to 45 minutes to complete, which would likely lead to increased respondent fatigue and lower response rates (26), significantly affecting the statistical power of the study. In contrast, shorter forms like the Ten-Item Personality Inventory (TIPI) are ideal for large-scale internet-based studies, where brevity is critical.

Short-form measurement instruments have been validated extensively. For example, Gosling et al. (2003) validated the TIPI using a dataset of over 300,000 respondents (27). Additionally, short forms such as the Mini-IPIP, BFI-S, and BFI-10 have been validated in large datasets such as the German Socio-Economic Panel (SOEP), a longitudinal study with over 20,000 participants (28, 29). These studies demonstrate that short forms can provide reliable assessments of the Big Five traits in large, diverse populations.

This trade-off between brevity and internal consistency is well-documented in the literature (16, 30, 31). Given the scope and scale of our study, we accepted this trade-off and TIPI was the most practical choice.

Also to address the Editor’s concerns we have added a new limitation to the study to recognise the trade-off between brevity and internal consistency.

Brief scale: TIPI is known as an ultra-brief scale with a trade-off between brevity and internal consistency. Use of ultra-brief scales does not provide the same level of depth as longer inventories and could introduce lower reliability than if a long-form personality test were used.

Further, we have added the following in the section considering future research (with appropriate references):

Future research should include further validation of short-form measurement of The Big Five Model instruments specifically in the multi-marathon population, potentially comparing them with longer measures such as 240-item NEO Personality Inventory, 44-item Big Five Inventory, 60-Item NEO Five-Factor Inventory or 100-trait Goldberg’s instrument (15, 26, 27, 28).

Additional new references to illustrate this point:

Specht, J., Egloff, B., & Schmukle, S. C. (2011). "Stability and change of personality across the life course: The impact of age and major life events on mean-level and rank-order stability of the Big Five." Journal of Personality and Social Psychology, 101(4), 862-882.

Rammstedt, B., & John, O. P. (2007). "Measuring personality in one minute or less: A 10-item short version of the Big Five Inventory in English and German." Journal of Research in Personality, 41(1), 203-212.

Gosling, S. D., Rentfrow, P. J., & Swann, W. B. (2003). "A very brief measure of the Big Five personality domains." Journal of Research in Personality, 37(6), 504-528.

**3) some lacking references and problems with the visibility of the figures.**

We also note the Editor’s comments on additional references and problems with the visibility of the figures. As per detail in Reviewer2’s comments, references have been rechecked and new references added appropriately throughout the background section.

Fig 1 has been validated by using the PLOS Pace figure checking tool and the converted TIFF file will be updated in the submission. Table 2 has been converted from an image to a native word table and Fig 2 has been converted from an image into a native word table and additionally transposed to ease understanding. All numbering and titles of figures and tables have been updated.

**4) You also need to address all minor suggestions of the reviewer.**

These have been addressed directly with the detail in the individual reviewer rebuttal comments.

By addressing the Editor's feedback, we believe the study's depth and rigour has been significantly improved.

We are grateful for the Editor’s and reviewers' helpful suggestions, which have helped us enhance the theoretical structure and overall quality of the submission.

**Reviewer 1 comments**

1) **Authors may benefit from further discussion regarding the application of both the Big Five Personality Model and Goal Setting Theory. Recent advancements in the understanding of human behavior indicate that various other theories and constructs can also elucidate behaviors related to physical activity.**

See answer to the editor comment 1. In addition to the Goal Setting Theory and the Big Five Personality Model we have incorporated Self-Determination Theory as part of the theoretical foundation for this submission further enriching the study’s theoretical depth, particularly as it pertains to behaviours related to physical activity.

2) **It remains ambiguous to me what substantial value is derived from involving 593 participants in a questionnaire grounded solely in these theories.**

We appreciate the reviewer's comment regarding the value of involving **593 participants** in a questionnaire grounded in the **Big Five Personality Model** and **Goal Setting Theory (GST)**.

The large sample size was a deliberate choice to ensure **statistical power**, **generalisability**, and the ability to conduct **subgroup analyses** that enrich the study's findings.

**Statistical Power and Generalisability**: The inclusion of 593 participants increases the **statistical power** of the study, allowing us to detect subtle effects of personality traits and goal-setting behaviours. Additionally, a larger sample provides more reliable and generalisable results, making our findings applicable to a broader population of endurance athletes across different ages, genders.

**Subgroup Analysis and Depth of Insight**: With a large sample, we were able to conduct detailed **subgroup analyses** (e.g., by gender and age group), offering deeper insights into how different personality traits and motivational patterns influence multi-marathoners’ performance. This depth of analysis would not be possible with a smaller sample size. In our case, we were able to apply the required significant statistical correction in our statistical analysis (Bonferroni statistical correction 50) while still getting significant results.

**Validation of Theories in multi-marathoners**: While both the Big Five Personality Model and Goal Setting Theory have been widely validated in other populations, their application to multi-marathoners and endurance athletes is relatively underexplored. This large sample allows us to provide important validation of these theories in a unique population, contributing to their broader application in sports psychology.

**Practical Applications**: The findings derived from this large sample can have significant practical applications. Coaches, sports psychologists, and athletes can benefit from understanding how personality traits and goal-setting behaviours influence endurance performance, leading to more personalised interventions and strategies to improve training and performance outcomes.

With the introduction of SDT the above points are further enhanced with a deeper theoretical foundation.

3) **It is important to consider the necessity of citations throughout the background section.**

Based on the reviewer’s comment, the background section has been enhanced and now has relevant references throughout.

4) **Concerns arise regarding the utilization of a questionnaire lacking prior validation for the specific population and language. While the presentation of the internal consistency and reliability of the survey is an important step, it concurrently underscores the shortcomings of the employed measures.**

We appreciate the reviewer’s concerns regarding the use of a questionnaire that lacks prior validation for this specific population and language. Indeed, the psychological analysis of multi-marathoning populations is largely absent from existing literature, which is one of the core gaps this study aims to address.

While no direct validation of short-form personality measures, such as TIPI, exists for multi-marathoners specifically, these tools have been extensively validated in endurance athletes and other sports contexts, which share similar physical and psychological challenges. For example, Zeiger and Zeiger (2018) used short-form personality assessments, including measures of mental toughness, in endurance athletes like ultra-marathoners. This population faces similar cognitive and emotional demands to multi-marathoners, demonstrating the applicability of these instruments.

Moreover, Laborde et al. (2016) used short-form personality measures to investigate positive personality-trait-like individual differences in athletes from both individual and team sports, further supporting the general use of short-form personality measures in athletic populations. Given the physical and psychological demands inherent in endurance sports, we believe TIPI is an appropriate and practical choice for this study.

This study thus contributes significantly to filling the gap in academic research regarding the psychology of multi-marathoning, while also adding to the existing body of literature that supports the use of brief personality instruments in sports. This point has been further detailed in response to editor comment 2.

**References supporting this point**:

- Laborde, S., Guillén, F., & Mosley, E. (2016). Positive personality-trait-like individual differences in athletes from individual- and team sports and in non-athletes. *Psychology of Sport and Exercise, 26*, 9–13. <https://doi.org/10.1016/j.psychsport.2016.05.009>
- Zeiger, J. S., & Zeiger, R. S. (2018). "Mental toughness latent profiles in endurance athletes." *PLOS ONE, 13*(2), e0193071. <https://doi.org/10.1371/journal.pone.0193071>

To address this point directly, we have added the following text into the **Introduction** with appropriate references.

While there has been limited psychological analysis of multi-marathon runners, short-form Big Five personality instruments have been validated in similar endurance sports populations. Studies by **Zeiger and Zeiger (2018)** used short-form personality measures to profile mental toughness in endurance athletes, such as ultra-marathoners, who face comparable physical and psychological demands. Similarly, **Laborde et al. (2016)** investigated personality traits in athletes using short form measures across both individual and team sports. These validations support the use of brief personality inventories like TIPI for capturing relevant psychological traits in multi-marathoning.

The survey was presented to respondents in multiple validated language translation. 78% were completed in English which is the base language for TIPI. Validated languages are available from the author of TIPI’s main web resource, each with validation reference(s). These validation references present a significant body of work that adds to the validation of TIPI across medium and large-scale populations. The advantage of having a short-form survey is that the translations are straight forward, and no issues were noted by the team regarding language. We do however acknowledge that subtle differences in interpretation may occur across different languages, even in validated versions. Therefore, we have added this as a **limitation**, noting that the study relied on validated translated versions of TIPI, which could introduce minor variability in responses. However, predominant use of English and use of validated translations and the straightforward nature and brevity of TIPI help mitigate this concern. Appropriate references of validated translations have also been added.

Language: A limitation of the study is the use of multiple validated translations in the survey. 78% of responses were completed in English (TIPI's base language). However, minor differences in interpretation across languages may introduce some variability. Predominant use of English, use of validated translations and the straightforward nature and brevity of TIPI mitigate this concern.

We agree that the presentation and internal consistency and reliability of the survey is an important step, and this study is the first time this has been achieved with the multi-marathon community. While highlighting the shortcomings of short form measurement instruments, we believe that previous validation by the TIPI authors in validating short form instruments in large diverse data sets and in complementary sports to multi-marathoning directly address this. This point is further explored in the rebuttal section 2 of the editors’ comments.

**Reviewer 2 comments**

**It offers highly relevant information about the personality traits of multi-marathon runners.
The data collection is extensive, with 593 responses, considering that this is a limited population, such as that of multi-marathon runners.**

**The statistical analyses are appropriate and very well conducted. They are carried out with an excellent level of detail, including the Bonferroni correction, which is not always performed.
Some results may be unexpected, such as in the dimension of openness to experience. However, for the rest of the results, the expected outcomes are essentially met.**

**In any case, the work provides an interesting perspective on the psychological characterization of multi-marathon runners. This is of great interest for practical intervention in counseling and psychological coaching for these athletes.**

**The main challenge of the work, as I understand it, lies in the use of the TIPI instrument to evaluate the Big Five personality dimensions. Although it is a sufficiently standardized and widely accepted tool by the scientific community in personality psychology.**

**In conclusion, this is a well-conceived and properly executed scientific study, offering relevant contributions to both the theory and practice of sports psychology.**

We appreciate the reviewers’ comments and see no actionable points in the comments. We do note the reviewers’ comments about the Big Five Personality Dimension being sufficiently standardised and widely accepted by the scientific community in personality psychology. This aligns completely with our assumptions and arguments in the submission and this rebuttal.
