# Supplementary material for: Unveiling the psychological traits of multi-marathoners: Insights from TIPI personality trait analysis
Source: PLoS One. 2025 Apr 1;20(4):e0311647. doi: 10.1371/journal.pone.0311647 (PMC11961011; doi:10.1371/journal.pone.0311647)
Supplement: Attachment — Submitted filename: Rebuttal TIPI FINAL 07.01.2025.docx [file pone.0311647.s002.docx]

**Editor: PLoS One**

***Title of the article:*** *Unveiling the Psychological Traits of Multi-Marathoners: Insights from TIPI Personality Trait Analysis.*

**Rebuttal document**

Thank you for the opportunity to revise our manuscript and for the detailed comments and suggestions provided by you and the reviewers. We appreciate why these comments were made and we have taken them all on board and have made substantial changes to the manuscript based on this feedback.

In general, we found comments supportive of the submission and appreciate the significant time and effort put in by the editor and reviewers to improve this study. We believe that this study is important not only to academics but also to the professionals and participants that engage with the sport of multi-marathoning. Below we address the concerns point by point and detail the revisions made in the manuscript.

This revised manuscript reflects a significant rework, incorporating substantial additional development and advanced statistical analysis. Specifically, the manuscript has been completely reframed in line with the editors suggestions. Key areas of enhancement include the introduction and integration of a 4th theoretical framework, the Health Belief Model (HMB), increased complexity and depth of the research questions and hypotheses, significant rework on the literary review, introduction of permutation-based methods and other statistical robustness methods, and the integration of Latent Class Analysis which is now pervasive throughout the manuscript. This has meant a significant rework of introduction, methods and results sections and a rewrite of the discussion and conclusion sections. We are confident that these extensive revisions have greatly strengthened the manuscript, addressing all the editor and reviewers comments comprehensively.

**Journal requirements.**

This revision of the submission meets PLOS ONE’s style requirements as outlined by PLOS ONE.

**Data Availability statement**

The entire data including any previously **published** data has been uploaded as supplementary files to this **submission**. This data is anonymised and can be used to replicate the results as presented in the submission.

**Author affiliation**

All authors’ affiliations reflect the institution where the study was carried out.

#### **1. Introduction and Literature Review:**

**Comment: Provide a more detailed review of the previous literature on athletes and Big Five personality traits, with a focus on how and why the personality profile of multi-marathoners may theoretically differ. Include a systematic analysis of previous research findings on this topic.**

**Response:** We acknowledge the importance of situating our study within the broader context of existing literature. While our initial systematic literary review of PubMed and Web of Science (not detailed in previous drafts) revealed over 12,000 articles on marathoning, we noted a significant gap in the literature regarding multi-marathoning. Recognising this and the theoretical overlap with endurance running, we have expanded the introduction to include details of this initial systematic literary review and also of previous research on Big Five personality traits among athletes and the relevance of the other theoretical frameworks used in analysis. This contextualises the study much better and lays a good framework for asking more detailed and searching research questions, which in turn much enhances the study.

#### 2. **Research Aim**

**Comment: The research aim is simplistic. Incorporate additional research questions and consider latent class analysis or including the entire sample and additional variables.  suggest incorporating additional research questions in both the literature review and analysis. For example, would it be feasible to include latent class analysis to examine variations within the population of interest? If possible, can you include the whole sample and other variables to expand your research questions?**

**Response:** We have rewritten the research aims and hypothesis to encompass more complex and searching questions, capturing variability within the multi-marathoner population. Specifically:

- In addition to the existing research aims, we have added new research aims and hypotheses that are drawn from the literary review and relevant theoretical frameworks. These new research aims and hypotheses are detailed in the Introduction.
- We introduced the Health Belief Model (HBM) as a new theoretical framework, providing insights into how psychological traits influence health-related behaviours and resilience in this population.
- Conducted LCA using the poLCA function from the R package poLCA to identify latent subgroups within our complete sample (TIPI results and previously published observational data), revealing latent patterns with common psychological profiles. This has revealed 4 significant latent subgroupings which are discussed and interpreted in the manuscript.
- Subsequently we have added a significant new hypothesis around subgroupings including those that are relevant to Latent Class Analysis (LCA)
- Described why and how we have integrated data from a previously published study which adds relevant variables to this study.

The Introduction, Methods, Results, Discussion and Conclusion sections have been expanded to reflect the new insights gained from this expanded approach.

The revised aims and hypotheses are now a lot more comprehensive and searching and offers a more comprehensive understanding of the psychological traits associated with multi-marathoning. These additions enrich the manuscript and address the editors’ request for a stronger theoretical foundation and adding additional research questions.

#### 3. **Statistical Analysis**

**Comment: While the statistical analysis addresses your aims, it is very basic by current standards of scientific rigor and novelty. I recommend including permutation-based methods to complement your research goals, along with non-parametric tests. You also investigate goal-setting behavior and report correlations with data from a previous observational study with a larger sample. However, the measures used in that comparison are not described in the Methods section, and the analysis remains highly descriptive. Also, the analysis needs to be better integrated with the rest of the paper. A more robust approach and statistical modeling, possibly including the entire sample, should be considered.**

**Response:**
We appreciate the feedback and have made significant enhancements to the statistical analysis to address these concerns.

**Permutation-Based Methods:**

To ensure robustness and address potential violations of parametric assumptions (e.g., normality and homoscedasticity), we incorporated **permutation-based methods** to complement non-parametric tests. Specifically, we:

- Performed permutation tests with **10,000 permutations** to empirically generate null distributions for test statistics.
- Calculated p-values by determining the proportion of permuted test statistics that were as extreme as or more extreme than the observed values.
- Reported these permutation-based p-values alongside standard non-parametric tests where appropriate, such as the Mann-Whitney U test and Spearman Rank Correlation tests.

Using **10,000 permutations** ensures precision and stability in p-value estimates, enhancing the reliability and robustness of our conclusions.

**Application Areas for Permutation-Based Methods:**

- Testing differences between multi-marathoners and the normative dataset.
- Correlating personality traits from this study with additional covariates from the dataset from the previously published observational study.

**Confidence Intervals for Correlation Analyses:**

In addition to Permutation-Based Methods, we have added **confidence intervals** to the correlation analyses to provide a clearer understanding of the precision and reliability of these estimates. This addition aligns with best practices for reporting statistical results and adds scientific rigour to the analysis.

**Integration of Observational Study Data:**

We have expanded the Methods section to provide a detailed description of the previous observational study’s data and measures, explaining why and how they were integrated into the current analysis. This includes:

- Demographic and goal-setting variables.
- Health conditions, dietary patterns, injuries, recoveries, and motivations

**Modelling Approaches:**

We have implemented LCA, revealing latent patterns with common psychological profiles. Subgrouping and in particular LCA groupings now form an integral part of this study and all areas have been changed to accommodate this.

**The entire data sample:**

In addition to the TIPI dataset from this study, also uploaded as part of this submission is the dataset from the previous published observational study on multi-marathoning. This will allow for anyone to replicate the analysis done in this study.

The Methods, Results, and Discussion sections have been revised to detail these enhancements and their implications. These revisions directly address the concerns raised, ensuring that our statistical analysis is robust, comprehensive, reproducible, and scientifically rigorous.

We believe these enhancements significantly strengthen the manuscript and demonstrate our commitment to providing reliable, reproduceable, and defensible results.

#### 4. **Presentation of Results**

**Comment: Improve the presentation of results. Avoid non-standard terms like “p < 0.00002 (extremely significant)” and use visualisations. Consider using figures to visualize your findings.**

**Response:**
We thank the editor for highlighting this important aspect, and we have made substantial revisions to the presentation of results to align with standard reporting practices. Including new visualisation figures and charts, to better illustrate our findings and enhance clarity. This includes a new radar chart visualising the comparison of TIPI data with TIPI norms and a LCA probability bar graph highlighting the proportion within each identified latent class.

#### **Changes Made**

- **Standardised Reporting of P-Values:**
  - We have replaced non-standard terms such as “p < 0.00002 (extremely significant)” with **p < 0.001**, following widely accepted conventions in statistical reporting.
  - Precise interpretations of statistical significance are provided without the use of subjective descriptors (e.g., “highly significant” or “extremely significant”).
- **Enhanced Clarity and Precision:**
  - Results are now presented with clear, concise statistical descriptions, including **effect sizes**, **confidence intervals**, and **p-values**, ensuring transparency and adherence to best practices.
  - Where relevant, **descriptive statistics** (e.g., means, standard deviations) accompany inferential statistics to provide context.
- **Structure and Flow:**
  - The Results section has been restructured for better readability, starting with descriptive statistics, followed by inferential analyses, and concluding with subgroup analyses and visualisations.

### **Revised Example from Results Section**

Instead of:

“TIPI Conscientiousness was significantly correlated with goal-setting behaviours, p < 0.00002 (extremely significant).”

The revised text reads:

TIPI Conscientiousness was positively correlated with goal-setting behaviours (Spearman’s ρ = 0.45, p < 0.001).

All tables have been updated to reflect this throughout the manuscript.

We believe these changes significantly improve the presentation of the results, ensuring they are both scientifically rigorous and accessible to a broad audience.

#### 5. **Methods Section**

**Comment: There is no mention of missing data or how it was handled, nor is it clear if any additional data was collected in your study. Describe all the measures of all variables.**

**Response:** The Methods section has been expanded to address missing data handling explicitly and describe the measures of all variables used in the study. These updates ensure transparency and reproducibility.

The following was added to the methods section.

Handling of Missing Data

The survey platform enforced data completion ensuring that all submitted responses were fully recorded. Only 100% completed surveys were used for analysis. Consequently, there were no cases of missing data, and all analyses were conducted on a complete dataset.

Measures of Variables are included throughout the methods sections as appropriate and reflect the following measure of variables.

- **Personality Traits**: Measured using the Ten Item Personality Inventory (TIPI) with the five personality traits calculated by reverse scoring.
- **Motivation, Injury, Recovery, and Diet**: Covariates were collected as multi-choice questions from a defined list.
- **Health Outcomes**: Participants self-reported whether or not they had specific conditions as an answer to binary questions (yes/no).
- **Demographics**: Demographic variables included age, gender, and the number of marathon completions. These were self-reported by respondents in fields that incorporated inherent number-checking to ensure accuracy and prevent invalid entries.

#### 6. **Study Limitations**

Comment: **Include a more critical and detailed overview of limitations, particularly in assessing only the Big Five personality traits and demographic variables without controlling for confounding variables.**

**Response:** The discussion now includes a more detailed overview of the study’s limitations. Specifically, we address the reliance on the Big Five personality traits and demographic variables and discuss the potential impact of unmeasured confounding variables. This critical assessment provides a balanced view of our findings.

Specifically, we have added the following limitations to the existing Limitations section.

**Measurement Instrument:** the study focused exclusively on the Big Five personality traits, as measured by the Ten Item Personality Inventory (TIPI). While TIPI is a validated and widely used tool for assessing personality, its brevity may limit the depth and granularity of the data compared to more comprehensive measures, such as the NEO-PI-R or BFI-44.

**Demographic variables:** were limited to age, gender, and the number of marathon completions. The study did not control for potentially confounding variables, such as socio-economic status, cultural background, or access to resources (e.g., coaching or training facilities), which could influence participation, personality expression, and health outcomes. These unmeasured variables may have introduced bias or limited the generalisability of the findings.

#### 7. **Suggestions for Further Research**

**Comment: Provide more substantive suggestions for theoretical development and future research.**

**Response:** We have expanded the discussion to include detailed suggestions for future research. These include exploring additional personality dimensions, utilising longitudinal designs, and investigating potential confounding variables.

The future research section has been rewritten and now includes.

Future Research Directions

Future research should explore how personality traits evolve with continued participation in multi-marathoning, using longitudinal studies to examine trait stability and dynamic changes over time. Such research could clarify whether resilience for example is a stable personality trait or a context-dependent capacity shaped by external challenges and support systems. Investigating the impact of specific interventions, such as tailored training programs, stress management techniques, and mental health support, could provide evidence-based strategies to enhance resilience and mitigate challenges associated with low emotional stability.

Expanding the diversity of study samples to include broader cultural, socioeconomic, and geographic contexts is essential for improving the generalisability of findings. This would also provide a more nuanced understanding of how external factors, such as access to resources or cultural attitudes, influence participation and personality expression in endurance sports.

Future studies should also validate and refine personality assessment tools, comparing brief measures like the Ten Item Personality Inventory (TIPI) to longer instruments, such as the NEO-PI-R. This would ensure the accuracy and reliability of personality measurement in multi-marathoners. Additionally, tracking subgroup dynamics identified through Latent Class Analysis (LCA) could reveal how health outcomes, motivation, and personality profiles evolve over time, informing tailored intervention strategies for distinct athlete profiles.

By addressing these areas, future research can advance understanding of the psychological and contextual factors that underpin multi-marathoning, providing a foundation for more effective support and interventions in this unique endurance sport.

#### 8. **Overall**

**Comment: Overall, I suggest a major reframing of the whole manuscript.**

We have completely reframed the whole manuscript. This is in line with the editors suggestions and also in line with the projects overall aims which until now was not concisely articulated in the manuscript. In particular we highlight that this reframing address gaps in academic research and documents the sport accordingly. It also highlights the theoretical frameworks used and the definition of specific personality driven subgroups through statistical analysis. This in turn provides insights into the sport and allows us to present recommendations to support the positives in these subgroupings and mitigate risks and vulnerabilities in other sub-groupings as appropriate.

To this end we have including this framing statement at the start of the introduction to better contextualise the manuscript and guide the reader quickly as to the over all aim of the study.

This study is framed as an academic exploration of multi-marathoning, documenting the sport while integrating established theoretical frameworks, including the Big Five Personality Model, Goal Setting Theory (GST), Self-Determination Theory (SDT), and the Health Belief Model (HBM). By adopting this framing, the research examines the psychological mechanisms driving multi-marathoners’ participation, health behaviours, and resilience. Through the identification of personality-driven subgroups, the study lays the groundwork for tailored intervention strategies aimed at enhancing well-being, sustaining participation, and addressing the distinctive challenges faced by this unique athletic community.

### 9. **Reviewers comments**

There are no actionable comments in the reviewers comments but believe that these enhancements are in line with previous reviewer comments.

### **Conclusion**

We believe the revisions have significantly improved the manuscript and addressed the concerns raised during the review process. We thank the editor for the constructive feedback, which has greatly strengthened our work, and we look forward to hearing your assessment of this revised version.

We are fully committed to this work and producing the best result for the multi-marathon community. Please do not hesitate to contact us if further clarification is required.

Sincerely,
