# Supplementary material for: Unveiling the psychological traits of multi-marathoners: Insights from TIPI personality trait analysis
Source: PLoS One. 2025 Apr 1;20(4):e0311647. doi: 10.1371/journal.pone.0311647 (PMC11961011; doi:10.1371/journal.pone.0311647)
Supplement: Attachment — Submitted filename: Rebuttal TIPI FINAL 14.02.2025.docx [file pone.0311647.s003.docx]

**Editor: PLoS One**

***Title of the article:*** *Unveiling the Psychological Traits of Multi-Marathoners: Insights from TIPI Personality Trait Analysis.*

**Rebuttal document**

Thank you for the opportunity to revise our manuscript and for the detailed comments and suggestions provided by Reviewer 3. We appreciate why these review comments were made and we have taken them all on board and have made appropriate changes to this new submission based on this feedback.

In general, we found comments supportive of the submission and appreciate the significant time and effort put in by the editor and reviewers to improve this study. We believe that this study is important not only to academics but also to the professionals and participants that engage with the sport of multi-marathoning. Below we address the concerns of Reviewer 3 point by point and detail the revisions made in the manuscript.

**Journal requirements.**

This revision of the submission meets PLOS ONE’s style requirements as outlined by PLOS ONE.

**Data Availability statement**

The entire data including any previously published data has been uploaded as supplementary files to this submission. This data is anonymised and can be used to replicate the results as presented in the submission.

**Author affiliation**

All authors’ affiliations reflect the institution where the study was carried out.

We will address Reviewer 3’s comments one-by-one.

#### **1. Keywords:**

**Comment: Change the keywords to other than in the title to ensure that your work is more recognizable in the science databases.**

**Response:** We appreciate that keywords play an important part in how this document is discovered in science databases and we have replaced the existing set of keywords. This should ensure that this work is more recognizable in science databases. These new keywords will be used as part of this new submission.

**Previous Keywords:** Marathon, Multi-marathon, running, marathon, Running, Psychology, psychology

**New Keywords:** Marathon, Multi-marathon, Endurance sports, Ultra-distance running, Marathon running, Personality traits, Psychological resilience, Goal setting in sports, Health and motivation, TIPI. Sport performance psychology, Psychological factors in endurance sports, Big Five personality traits, Self-determination in sports.

#### **2. Abstract abbreviation:**

**Comment: Abbreviate the abstract to relevant issues, the current form is overloaded.**

**Response**: We appreciate the reviewers comments and agree with the comment. We have rewritten the abstract so that it is abbreviated and removes unnecessary details while maintaining essential findings. It now directly presents the key points without overloading with excessive methodology or result details. The authors believe this now reads much better and is more concise and thanks Reviewer 3 for their comment.

The new abstract now reads as follows:

**Abstract**

**Objectives:** Multi-marathoners, athletes dedicated to completing 100+ marathons, represent a unique endurance sport subculture. This study examines their psychological traits using the Ten Item Personality Inventory (TIPI) and Latent Class Analysis (LCA) to identify personality-based profiles and subgroup differences.

**Methods:** An online cross-sectional survey of 593 multi-marathoners (56% men, 44% women, mean age = 53.87, SD = 9.91, countries =22) collected TIPI data. Reliability was assessed using Cronbach’s Alpha and Guttman’s Lambda 6. Statistical analyses included Mann-Whitney U tests, ANOVA Aligned Rank Transform (ART), Wilcoxon post-hoc tests, and Spearman’s correlations to examine personality differences across gender, age and health variables. LCA identified distinct personality subgroups. Normative TIPI data served as a comparison benchmark.

**Results:** Multi-marathoners exhibited higher conscientiousness (F(1,591) = 2.42, p <0.001) but lower emotional stability (F(1,591) = 5.525, p<0.001) than the general population, suggesting strong goal-directed behaviour but challenges in stress management. Women showed higher agreeableness (W = 50809, p<0.00091), while age-related differences were not statistically significant. LCA revealed four personality-based subgroups, including those with high resilience and others with health vulnerabilities.

**Conclusion:** Multi-marathoners display distinct psychological traits, particularly high conscientiousness and lower emotional stability. These findings highlight the need for tailored psychological interventions to support multi-marathon athletes’ participation and well-being. Future research should explore longitudinal patterns and explore the efficacy of psychological interventions to enhance participation and well-being.

#### **3. Introduction :**

**Comment: The Introduction should be abbreviated by not capturing the cited research, but aptly formulating the content without embellishments.**

**Response**: We appreciate the reviewer’s feedback on the Introduction and acknowledge the need for a more concise presentation. In response, we have made the following revisions:

1. **Condensed discussions on theoretical frameworks** (e.g., Big Five Personality Model, Goal Setting Theory, Self-Determination Theory, Health Belief Model) to essential points, ensuring they are succinctly integrated into the research context without embellishment.
2. **Removed detailed summaries of cited research** and instead provided concise references to key studies without excessive description.
3. **Eliminated redundant explanations and embellishments** to maintain a clear and direct narrative leading to the research questions and hypothesis.

We are cognisant that the Introduction has previously been the subject of other reviewers comments, and these modifications enhance the clarity and focus of the Introduction while maintaining the necessary context for our study and the integrity of all reviewers comments. In particular, we believe these refinements described above align with the reviewer 3’s recommendation and improve the overall readability of the manuscript.

#### **4. Results:**

**Comment: Results, please improve so that they are only perceptive sentences.**

**Response**: We appreciate Reviewer 3’s feedback and have revised the Results section to ensure that it consists solely of perceptive and objective statements. We have removed any interpretative or speculative language and focused on presenting the findings clearly and concisely. The revised text now strictly describes the observed data, statistical outcomes, and trends without unnecessary discussion or inference.

We believe these revisions enhance the clarity and scientific rigor of the manuscript. We appreciate the reviewer’s insights and trust that the updated Results section now meets the required standards.

#### **5. Table abbreviations**

**Comment: Explanations of the abbreviations used should be added under the tables.**

**Response**: We appreciate the reviewer’s feedback and have implemented the requested changes by adding explanatory footnotes directly below each table. These footnotes provide definitions for all abbreviations used within the respective tables, ensuring that readers can immediately understand the terminology without needing to refer to other sections of the manuscript.

To ensure clarity, we have formatted the footnotes consistently across all tables, using a "Note" section directly beneath each table. For example:

Note: M = Mean, SD = Standard Deviation, p-val = p-value, ρ = Spearman’s correlation coefficient, Pr = Probability.

#### **6. Multiplicity of subsections**

**Comment: There is a multiplicity of subsections, which should be arranged in Discussion, Limitation, Directions for further research, Practical implications.**

**Response**: We appreciate the reviewer 3’s guidance on structuring the manuscript and have revised the organisation to ensure a clearer and more systematic presentation. The key subsections have now been arranged as follows:

1. **Discussion** – This section integrates the key findings with relevant literature, providing a cohesive interpretation of the results.
2. **Limitations** – We have created a dedicated subsection outlining the study’s constraints, including methodological considerations, potential biases, and data limitations.
3. **Directions for Further Research** – All recommendations for future research avenues have been consolidated into this section to provide clear guidance on how the study can be expanded or extended.
4. **Practical Implications** – This subsection explicitly outlines the real-world relevance of the findings, particularly in the context of multi-marathon participation, personality traits, and associated health factors.

Regarding the **Ethical Considerations** and **Research/Policy Implications** subsections, we have carefully evaluated their placement:

- **Ethical Considerations** remains a separate section, as it is a critical part of research transparency and is often standard in manuscripts. The authors believe that it makes sense that it remains a separate subsection.
- **Research/Policy Implications** has been integrated into the **Practical Implications** section. The policy-related insights naturally align with the broader discussion on practical applications, ensuring a streamlined and logical structure.

These revisions enhance the manuscript’s readability and alignment with academic conventions while preserving essential information. We appreciate this valuable feedback and believe these changes improve the clarity and impact of the manuscript.

#### **7. Conclusion**

**Comment: Conclusions are convictions about the veracity of your results, not a summary. This should be corrected.**

**Response**: We appreciate the reviewer’s feedback and have revised the Conclusion section to ensure it reflects convictions about the veracity of our results, rather than serving as a summary.

Changes Implemented:

- The Conclusion now explicitly states the robustness and reliability of our findings, emphasising how the results contribute to existing knowledge.
- Any content that resembled a summary of the study has been reworded to highlight the strength and implications of our findings.
- The section now addresses the validity of the methodology and statistical rigor, reinforcing the reliability of the conclusions drawn.

These revisions ensure that the Conclusion is aligned with academic standards by presenting well-founded assertions based on the study’s evidence, rather than a restatement of findings.

We appreciate this valuable feedback and believe the revised Conclusion better supports the integrity of our research.

#### **7. References**

**Comment: *References should be at most 10 years back, i.e., 2015. This should be corrected. I can suggest known papers on research on personality traits in sports. Consider referring to:***

- **DOI: 10.1519/JSC.0000000000002376**
- **DOI: 10.1016/j.paid.2020.109973**
- **DOI: 10.1002/brb3.2145**
- **DOI: 10.3389/fpsyg.2023.1284378**
- **DOI: 10.15561/20755279.2024.0302**
- **DOI: 10.3389/fpsyg.2024.1428107**

**Response** We sincerely appreciate the reviewer’s suggestion to update our references to ensure they are within the last 10 years and reflect recent research developments in personality traits and endurance sports. In using the suggested references we have found them extremely valuable in strengthening the manuscript by providing contemporary empirical support for key arguments.

In addition to incorporating the suggested references, we also reviewed all existing citations and made updates where necessary to ensure they meet the 10-year threshold (2015-present). Below, we outline (A) how we integrated the six suggested references and (B) how we revised our use of existing references to ensure alignment with the latest research were possible.

**(A) How We Used the Suggested References:**

We carefully reviewed each reference and integrated them into the manuscript in a manner that strengthens the theoretical foundation, supports our hypotheses, and enhances the discussion of personality traits in multi-marathoners. Below, we outline where each reference was incorporated and its contribution:

1. Piepiora, 2021 (DOI: 10.1002/brb3.2145)

Where Used: Introduction
How It Strengthened the Manuscript:

- This study was instrumental in reinforcing the distinctive personality profiles of endurance athletes.
- It supports our argument that multi-marathoners, like elite athletes, exhibit personality traits associated with resilience, perseverance, and conscientiousness.

Manuscript Revision:

*"The defining achievement for many multi-marathoners is reaching the milestone of completing 100 marathons, a feat that requires extraordinary dedication, perseverance, and resilience (Piepiora, 2021)”*

2. Shuai et al., 2023 (DOI: 10.3389/fpsyg.2023.1284378)

Where Used: Introduction
How It Strengthened the Manuscript:

- Provided a systematic review of personality traits in athletes, demonstrating that conscientiousness is positively associated with endurance sports success, while emotional stability is linked to stress and burnout.
- Helped justify the inclusion of conscientiousness and emotional stability in our hypothesis.

Manuscript Revision:

*"Endurance athletes often experience significant psychological challenges, including heightened stress, anxiety, and depressive symptoms, which can impact both performance and sustained participation (Shuai et al., 2023)”*

3. Piepiora et al., 2024 (DOI: 10.3389/fpsyg.2024.1428107)

Where Used: Discussion
How It Strengthened the Manuscript:

- Provided recent evidence on personality subgroups in competitive and elite athletes, reinforcing our argument that latent personality clusters exist in multi-marathoners.
- Helped support our use of Latent Class Analysis (LCA) in the study.

Manuscript Revision:

*"The identification of latent classes adds depth to the findings, revealing how subgroup-specific patterns of personality and health influence participation behaviours. This aligns with recent research demonstrating that elite and competitive athletes exhibit distinct personality clusters, reinforcing the importance of tailored psychological interventions (Piepiora et al., 2024)”*

4. Piepiora, 2024 (DOI: 10.15561/20755279.2024.0302)

Where Used: Practical Implications
How It Strengthened the Manuscript:

- Helped strengthen our goal setting and motivation discussion by linking conscientiousness and emotional stability with long-term endurance sports engagement.

Manuscript Revision (Practical Implications):

*"Athletes high in conscientiousness and emotional stability are more likely to sustain long-term training and competition, while openness and extraversion influence motivation and engagement (Piepiora, 2024)”*

5. Sutin et al., 2020 (DOI: 10.1016/j.paid.2020.109973)

Where Used: Introduction
How It Strengthened the Manuscript:

- Provided strong empirical support for using the Big Five Personality Model in endurance athletes.
- Helped reinforce our rationale for focusing on conscientiousness and emotional stability in our hypothesis.

Manuscript Revision:

*"Trait theory categorises personality into five core dimensions: openness, conscientiousness, extraversion, agreeableness, and emotional stability (neuroticism) (Sutin et al., 2020)”*

6. Stine et al., 2019 (DOI: 10.1519/JSC.0000000000002376)

Where Used: Practical Implications
How It Strengthened the Manuscript:

- Supported our argument on goal-setting theory in endurance sports, reinforcing how structured goals enhance performance.
- Helped strengthen the link between goal setting and personality traits in multi-marathoners.

Manuscript Revision:

*"Goal setting plays a crucial role in endurance sports, with effective self-regulation and structured goals linked to enhanced performance and resilience (Stine et al., 2019)”*

**(B) Revisions to Existing References**

We also reviewed and updated all citations to ensure they were within the last 10 years (2015-present). The following actions were taken:

- Replaced older references (pre-2015) with recent, high-impact studies where possible.
- Retained essential older references where they remain foundational and could not be replaced.

Below is a list of those references that breach the 2015 rule. Included is either a justification for continued inclusion or replacement references.

| **Citation** | **Justification for Inclusion** | **Potential Replacement** |
| --- | --- | --- |
| John OPS, S. (1999). The Big Five trait taxonomy: History, measurement, and theoretical perspectives. | Foundational work on Big Five personality traits. |  |
| Allport GW. (1961). Pattern and Growth in Personality. | Seminal work in personality theory. |  |
| Cattell RB. (1946). The Description and Measurement of Personality. | One of the earliest works on personality trait measurement. |  |
| Eysenck HJ. (1947). Dimensions of Personality. | Key contribution to personality theory, precursor to Big Five. |  |
| Deci EL, Ryan RM. (1985). Intrinsic motivation and self-determination in human behavior. | Core theory for self-determination, widely applied in sports psychology. | ('Ryan RM, Deci EL. (2017). Self-determination theory: Basic psychological needs in motivation, development, and wellness.', 'Updated book expanding on SDT applications.') |
| Rosenstock IM. (1990). The health belief model: Explaining health behavior through expectancies. | Foundational model for understanding health behaviors. | ((2015). The Health Belief Model. In Glanz K, Rimer BK, Viswanath K (Eds.), Health Behavior: Theory, Research, and Practice (5th ed.). Jossey-Bass.', 'More recent review of Health Belief Model applications.') |
| Costa PT, Jr., McCrae RR. (2008). The Revised NEO Personality Inventory (NEO-PI-R). | Key assessment tool for Big Five personality traits. |  |
| McCrae RR, Costa PT Jr. (2008). A five-factor theory of personality. | Comprehensive theory on Big Five personality structure. |  |
| Robins RW, Tracy JL, Trzesniewski KH, Potter J, Gosling SD. (2001). Personality correlates of self-esteem. | Research on the relationship between personality and self-esteem. Core manuscript with TIPI founder as author |  |
| Locke EA, Latham GP. (2005). Goal Setting Theory: Theory building by induction. | Major work on goal-setting theory, widely cited in sports and motivation research. | ('Locke EA, Latham GP. (2019). Advances in Goal Setting Theory. Journal of Business and Psychology, 34(1), 19-31.', 'Updated review of Goal-Setting Theory applications.') |
| Gosling SD, Rentfrow PJ, Swann WB Jr. (2003). A very brief measure of the Big-Five personality domains. | Development of TIPI, which is used in this study. |  |
| Nunnally JC, Bernstein IH. (1994). Psychometric Theory. | Classic book on psychometrics, foundational in measurement theory. | ('DeVellis RF. (2017). Scale Development: Theory and Applications (4th ed.). Sage.', 'More recent reference for psychometrics and measurement theory.') |
| Conover WJ. (1999). Practical Nonparametric Statistics. | Standard reference for nonparametric statistical methods. | ('Gibbons JD, Chakraborti S. (2020). Nonparametric Statistical Inference (6th ed.). Chapman & Hall.', 'Updated statistical reference for nonparametric methods.') |
| Maxwell SE, Delaney HD. (2004). Designing Experiments and Analyzing Data: A Model Comparison Perspective. | Key reference for experimental design and statistical analysis. | ('Montgomery DC. (2020). Design and Analysis of Experiments (10th ed.). Wiley.', 'More recent work on experimental design and data analysis.') |
| Field A. (2013). Discovering Statistics Using IBM SPSS Statistics. | Widely used statistical reference, practical for modern research. |  |
| Ingledew DK, Markland D. (2008). The role of motives in exercise participation. | Research on motivation and sports participation. | ('Roberts BW, Luo J, Briley DA, Chow PI, Su R, Hill PL. (2017). A systematic review of personality trait change through intervention. Psychological Bulletin, 143(2), 117-141.', 'More recent work on personality and exercise motivation.') |
| Specht J, Egloff B, Schmukle SC. (2011). Stability and change of personality across the life course. | Widely used Longitudinal study on personality trait stability over time. |  |
| Rammstedt B, John OP. (2007). Measuring personality in one minute or less: A 10-item short version of the Big Five Inventory. | Development of short-form Big Five personality assessments. |  |
| Oshio A, Abe S, Cutrone P. (2012). Development, reliability, and validity of the Japanese Version of the Ten Item Personality Inventory (TIPI-J). | Validation of TIPI in different languages, relevant for cross-cultural research. |  |
| Renau V, Oberst U, Gosling SD, Rusiñol J, Chamarro A. (2013). Translation and validation of the Ten-Item Personality Inventory into Spanish and Catalan. | Cross-cultural validation of TIPI, demonstrating its global applicability. |  |

By replacing the older references with newer citations we ensure that the newest research is always used when appropriate.

We sincerely appreciate the reviewer’s insightful recommendations, which have significantly enhanced the depth, credibility, and contemporary relevance of our study.

**Conclusion**

We believe the revisions have significantly improved the manuscript and addressed the suggestions raised by Reviewer 3. We thank the editor and review team for the constructive feedback, which has greatly strengthened our work, and we look forward to hearing your assessment of this revised version.

We are fully committed to this work and producing the best result for the multi-marathon community. Please do not hesitate to contact us if further clarification is required.

Sincerely,
